# Endothelial Progenitor Cells May Be Related to Major Amputation after Angioplasty in Patients with Critical Limb Ischemia

**DOI:** 10.3390/cells12040584

**Published:** 2023-02-11

**Authors:** Daniel Santillán-Cortez, Eduardo Vera-Gómez, Alejandro Hernández-Patricio, Atzín Suá Ruíz-Hernández, Juan Ariel Gutiérrez-Buendía, Karen De la Vega-Moreno, Yasser Alberto Rizo-García, Oscar Antonio Loman-Zuñiga, Ignacio Escotto-Sánchez, Juan Miguel Rodríguez-Trejo, Mario Antonio Téllez-González, Christian Gabriel Toledo-Lozano, Tania Ortega-Rosas, Silvia García, Paul Mondragón-Terán, Juan Antonio Suárez-Cuenca

**Affiliations:** 1 Experimental Metabolism and Clinical Research Laboratory, Clinical Research Department, Division of Biomedical Research, Centro Médico Nacional “20 de Noviembre”, Instituto de Seguridad y Servicios Sociales para los Trabajadores del Estado, Mexico City P.O. 03100, Mexico; 2 Vascular Surgery and Angiology Department, Centro Médico Nacional “20 de Noviembre”, Instituto de Seguridad y Servicios Sociales para los Trabajadores del Estado, Mexico City P.O. 03100, Mexico; 3 Regenerative Medicine and Tissue Engineering Laboratory, Coordination of Research, Centro Médico Nacional “20 de Noviembre”, Instituto de Seguridad y Servicios Sociales para los Trabajadores del Estado, Mexico City P.O. 03100, Mexico

**Keywords:** endothelial progenitor cells (EPCs), lower limb amputation, critical limb ischemia (CLI), peripheral artery disease (PAD)

## Abstract

Background: Critical limb ischemia represents an advanced stage of peripheral arterial disease. Angioplasty improves blood flow to the limb; however, some patients progress irreversibly to lower limb amputation. Few studies have explored the predictive potential of biomarkers during postangioplasty outcomes. Aim: To evaluate the behavior of endothelial progenitor cells in patients with critical limb ischemia, in relation to their postangioplasty outcome. Methods: Twenty patients with critical limb ischemia, candidates for angioplasty, were enrolled. Flow-mediated dilation, as well as endothelial progenitor cells (subpopulations CD45+/CD34+/CD133+/CD184+ and CD45+/CD/34+/KDR[VEGFR-2]+ estimated by flow cytometry) from blood flow close to vascular damage, were evaluated before and after angioplasty. Association with lower limb amputation during a 30-day follow-up was analyzed. Results: Endothelial progenitor cells were related with flow-mediated dilation. A higher number of baseline EPCs CD45^+^CD34^+^KDR^+^, as well as an impaired reactivity of endothelial progenitor cells CD45^+^CD34^+^CD133^+^CD184^+^ after angioplasty, were observed in cases further undergoing major limb amputation, with a significant discrimination ability and risk (0.75, specificity 0.83 and RR 4.5 *p* < 0.05). Conclusions: Endothelial progenitor cells were related with endothelial dysfunction, whereas a higher baseline number of the subpopulation CD45^+^CD34^+^KDR^+^, as well as an impaired reactivity of subpopulation CD45^+^CD34^+^CD133^+^CD184^+^ after angioplasty, showed a predictive ability for major limb amputation in patients with critical limb ischemia.

## 1. Introduction

Peripheral arterial disease (PAD) is a multifactorial, chronic, and progressive disease characterized by vascular lesions and a different degree of stenosis in the arteries responsible for blood supply, particularly to the body extremities [1,2,3]. PAD affects around 10% of the population older than 70 years of age in the USA; while up to 7% of such cases are submitted to lower limb amputation [4,5]. Mexico shows a slightly higher prevalence of PAD than European populations, but lower than Asian populations [6,7].

The most consistent risk factors associated with PAD are (1) race—an ankle to brachial index (ABI) ≤0.90 is more common among non-Hispanic blacks than whites; (2) sex—the prevalence of PAD, symptomatic or asymptomatic, is slightly higher in men than in women, particularly in younger age groups; (3) diabetes mellitus—intermittent claudication is twice as common among diabetic patients than among nondiabetic patients. Furthermore, PAD in diabetic patients is more aggressive compared to nondiabetics, with involvement of larger vessels together with the participation of distal symmetric neuropathy; (4) systemic arterial hypertension—there is an association between hypertension and PAD, particularly with systolic blood pressure. It has been suggested that almost one-third of the risk of claudication may be attributable to blood pressure > 160/100 mmHg; (5) dyslipidemia—it has been observed that a fasting cholesterol level > 270 mg/dL is associated with a doubling of the incidence of intermittent claudication and is an independent risk factor for PAD; 6) chronic kidney disease—the prevalence of PAD has been estimated up to 38% for noncompressible ABI cases; (7) smoking—considered the most important risk factor for PAD. In fact, most of diagnosis of PAD in smokers are made a decade earlier than nonsmokers, and its severity tends to increase according to the number of cigarettes smoked, whereas smoking cessation among patients with claudication has been shown to improve various related functional and physiologic measures, as well as reduce mortality [3,6,7,8,9,10,11].

In addition, some circulating proinflammatory mediators have been identified as significantly related with development of PAD, such as increased serum concentration of fibrinogen, C-reactive protein, and homocystein, which have been associated with PAD in several studies [1,2,3] 

The clinical scenario of PAD can range from the asymptomatic stage to critical limb ischemia (CLI) [2], which is the most serious presentation characterized by pain at rest, ulcers, or gangrene attributable to occluded arteries and vascular involvement. Prognosis is quite poor, with a prevalence of major amputation and one-year mortality of 30% and 25%, respectively [12,13]. Angioplasty is a minimally invasive and commonly used method, capable of re-establishing blood flow in the area with a vascular injury in cases of PAD with CLI [5]; however, some patients may require further major amputation of the lower limb, despite previous endovascular therapy. Therefore, it is important to explore new methods for early identification of unfavorable outcomes in patients with CLI undergoing angioplasty.

PAD is characterized by a diffuse and progressive process affecting morphology and function of blood vessels. Although its etiology is unknown, the most consistent proposed mechanisms refer to an inflammatory process as well as the response to lipid deposition in the arterial wall. Pathophysiologically, PAD is related to endothelial damage and the triggering of inflammatory mediators such as IL-1β; followed by continuous attempts of endothelial regeneration denoted by the release of molecules such as ICAM-1 (Intercellular Adhesion Molecule 1) and VEGFR (Vascular Endothelial Growth Factor Receptor) [1,2,14]. The limited capacity of endothelial repair leads to atherosclerotic lesions. The atherogenic process arises when endothelial damage is sufficient to induce the internalization of low-density lipoproteins (LDL) into the subendothelial layer, where they are oxidized. The resulting cell, with a high lipid content, is called a spongy cell or "foam cell". Subsequently, the aggression of different injurious agents on the arterial wall will alter the homeostatic properties of the endothelium, increasing the adhesion and permeability of leukocytes and platelets, losing anticoagulant properties. The result will be the growth of the plaque due to an increase in the extracellular matrix, accumulation of "foam cells", smooth muscle cells, the formation of thrombi due to platelet aggregation, and the appearance of foci of necrosis in the plaque, contributing with blood flow occlusion and emboligenic potential related with the progression of PAD severity [7,12,13,15]. 

Nowadays, the participation of cellular populations with endothelial repair properties migrating towards atherosclerotic plaque has been increasingly recognized. The first evidence indicating the presence of endothelial progenitor cells (EPCs) in adult circulation arose in 1997, when CD34+ cells were isolated from human peripheral blood and differentiated in vitro into cells with endothelial characteristics expressing endothelial markers such as CD31, E-selectin, and vascular endothelial growth factor receptor 2 (VEGFR-2), also called receptor kinase insertion domain (KDR) or Flk-1. Furthermore, it was described that CD34+/Flk-1+ cells were capable of inducing neovascularization. The discovery of EPCs in peripheral blood and its claimed involvement in the formation of new blood vessels revolutionized the field of vascular biology [12]. The characteristic immunophenotypic markers of EPCs are CD34, CD133, KDR (VEGFR-2), Tie-2, and the lectin ligand UEA-1; however, to date there is no marker that can discriminate whether the source is endothelial cells or endothelial progenitor cells [12,14] 

EPCs have high proliferative and differentiation capacity, whereas other mechanisms such as EPCs’ migration to the systemic circulation, vascular adhesion, and/or maturation are still the subject of several studies, and it has been speculated whether EPCs promote endothelial repair and angiogenesis in response to tissue ischemia. Moreover, the maturation and differentiation process of EPCs are important mechanisms involved in vessel repair. EPCs are sourced from immature progenitor cells derived from the bone marrow. These immature cells possess the ability to proliferate, migrate and differentiate into different cells, including EPCs, further becoming mature endothelial cells in the blood vessel wall [16,17,18,19].

Modulation of blood vessels formation and repair are important processes during all stages of life. In the embryonic period, the circulatory system is formed by the process known as vasculogenesis, while in adulthood, vessel formation and repair are produced by angiogenesis, a process that involves new vessel formation from pre-existing adjacent blood vessels. Both vasculogenesis and angiogenesis involve the mobilization of EPCs from the bone marrow to the target organs, where EPCs eventually differentiate into mature endothelial cells [20,21,22,23]. This highlights the role of EPCs to maintain vascular homeostasis through endothelial restoration mechanisms.

In the other hand, normal arterial function depends on the complex interaction between molecules derived from the vascular endothelium and the environment. In this sense, the term endothelial dysfunction (ED) refers to a loss of vascular homeostasis, where impaired vasoactive, anticoagulant, anti-inflammatory properties, and dysregulated vascular remodeling of growth result from a loss of nitric oxide bioactivity in the endothelium. Such a reduction in nitric oxide activity can be caused by (1) decreased eNOS (nitric oxide synthase 3 [NOS3]) expression; (2) insufficient substrate (L-arginine) or cofactors (tetrahydrobiopterin or BH4) for eNOS or the presence of antagonists (e.g., dimethyl asymmetric arginine); (3) impaired eNOS activation (e.g., caused by increased caveolin); and (4) increased NO degradation rate. The last two causes are associated with reactive oxygen species (ROS), such as the superoxide radical and the production of the potentially toxic peroxynitrate. The main sources of ROS in vascular cells include xanthine oxidase, NADH/NADPH oxidase, and eNOS [8,9].

It is also known that ED correlates with increased serum markers (i.e., adhesion molecules, selectins, C-reactive protein, MDA, IL-6) as well as with angiographically demonstrated coronary endothelial dysfunction. It has been claimed that ED predisposes to vasoconstriction, thrombosis, and atherosclerosis; therefore, the presence of ED indicates "preclinical" vascular disease, and may potentially identify patients in whom therapeutic intervention might be beneficial. Furthermore, ED may be evaluated by vascular reactivity measures such as FMD (flow-mediated dilation) and ABI, and severely affects patients with CLI [24,25,26].

In a previous study from our group, the translational relevance of circulating mononuclear progenitor cells in coronary vascular injury and repair was evidenced. We characterized the ability of EPCs to predict major adverse cardiovascular events (MACEs) and their influence in the prognosis of patients with coronary artery disease undergoing coronary angioplasty. We observed fewer EPCs in the population that presented MACEs, whereas more significant changes were observed in coronary endothelial progenitors CD45/CD34/CD133 and CD45/CD34/CD133/184 than other EPC subpopulations. Therefore, it is conceivable that differentiation ability and/or angioreparative potential between EPC subpopulations have clinical potential implications. Likewise, coronary circulating EPCs showed higher prognostic ability than their peripheral distribution. Only a few studies have evaluated the potential of coronary sampling for heart diseases. Consistently with our results, interesting differences with peripheral sampling and more precise predictive performance of coronary biomarkers have been evidenced, suggesting the relevance of sampling location in the evaluation of prognostic biomarkers in coronary artery disease. To our knowledge, this is the first study prospectively evaluating coronary and peripheral circulating nonsoluble EPCs and soluble mediators, and their role as prognostic biomarkers, in population with coronary artery disease submitted to coronary angioplasty and stenting [27].

Currently, there are few studies regarding outcome predictors after angioplasty in CLI, so this study aimed to evaluate the role of EPCs in the prediction of major limb amputation after angioplasty in patients with CLI.

## 2. Materials and Methods

### 2.1. Design

Quasi-experimental cohort, prospective study with discriminative ability analysis.

### 2.2. Study Population

Male patients who attended the Angiology and Vascular Surgery Department of the National Medical Center “20 de Noviembre”, Instituto de Seguridad y Servicios Sociales de los Trabajadores del Estado (ISSSTE), during the period from February 2016 to October 2016. Patients were diagnosed with CLI and were candidates for the endovascular procedure of balloon angioplasty. All patients received treatment with platelet antiaggregation and/or anticoagulation, statins, antihypertensive, and metabolic control drugs, according to international guidelines. They were excluded if they had crossover procedure, iliac stenosis, abdominal aortic or aortic coarctation, heart failure NYHA III or IV, severe cardiac arrhythmia, malignancies, or comorbidities related to significant inflammatory processes, or were receiving anti-inflammatory treatment (steroids, immunomodulators, or cytotoxics). 

### 2.3. Clinical Demographic Characteristics

Clinical demographic characteristics included age, smoking status, and comorbidities such as diabetes mellitus, systemic arterial hypertension, dyslipidemia, and others. Diabetes mellitus was defined according to the guidelines from the American Diabetes Association with one of the following conditions (repeated for confirmation at a separate date): (1) Hb > A1C 6.5%, (2) fasting glucose > 126 mg/dL, or (3) 2-h plasma glucose > 200 mg/dL during an oral glucose tolerance test. Systemic arterial hypertension was defined as blood pressure > 140/90 mmHg. Dyslipidemia was defined as 1 or more of the following conditions: (1) total cholesterol > 200 mg/dL, (2) LDL cholesterol > 100 mg/dL, (3) HDL cholesterol. 

The project was approved by the Research, Biosafety and Ethics Committee of the National Medical Center “20 de Noviembre”, ISSSTE, ID 294.2016. Their approach was based on the Declaration of Helsinki, and all patients gave their informed consent to participate.

### 2.4. Evaluation of Vascular Damage

The severity of vascular damage was assessed using the following scores: Rutherford [1] (Stage 0—asymptomatic; Stage 1—mild claudication; Stage 2—moderate claudication; Stage 3—severe claudication: Stage 4—rest pain; Stage 5—ischemic ulceration, not exceeding ulcers of the digits of the foot; Stage 6—severe ischemic ulcers or frank gangrene), as well as the Wound, Ischemia, foot Infection (WIFI) classification system from the Society for Vascular Surgery [1] (Wound (W) component: 0—no ulcer or gangrene (ischemic pain at rest); 1—small or superficial ulcer on leg or foot, without gangrene, either simple digital amputation or skin coverage; 2—deep ulcer with exposed bone, joint, or tendon ± gangrene limited to digits (multiple digital amputations or standard transmetatarsal amputation ± skin coverage); 3—deep, extensive ulcer involving forefoot and/or midfoot ± calcaneal involvement ± extensive gangrene (complex reconstruction of the foot or nontraditional transmetatarsal amputation). Ischemia (I) component, considering ankle-to-brachial index (ABI): 1 = ≥ 0.80; 2 = 0.6–0.79; 3 = 0.4–0.59 ≤ 0.39. If systolic blood pressure of the ankle is considered: 1 = > 100 mmHg; 2 = 70–100 mmHg; 3 = 50–70 mmHg < 50 mmHg. If transcutaneous oxygen pressure is considered: 1 = ≥ 60 mmHg; 2 = 40–59 mmHg; 30–39 mmHg < 30 mmHg. Foot Infection (fI) component: 0—uninfected; 1—mild local infection, involving only the skin and subcutaneous tissue, erythema > 0.5 to ≤ 2 cm.

### 2.5. Endothelial Dysfunction and Atherogenesis

The degree of endothelial dysfunction was evaluated by flow-mediated dilation (FMD) test, according to the recommendations of the “International Brachial Artery Reactivity Task Force”, using a Hitachi ultrasound equipment, Aloka Prosound Alpha-7, and vascular linear transducer of 5–10 Mhz. Briefly, the diameter of the brachial artery was determined above the antecubital fossa. Subsequently, the cuff of the sphygmomanometer was placed below the measurement site in the forearm and insufflated at 50 mmHg above the systolic blood pressure for 5 min and then deflated; then, the diameter of the brachial artery was redetermined within the next 60 s. The degree of dilatation (DD) was calculated using the following equation:

$$\mathrm{DD}\left(\%\right)=\frac{\left(\left(\mathrm{maximum}\;\mathrm{diameter}\;\mathrm{after}\;\mathrm{transient}\;\mathrm{ischemia}\;-\mathrm{basal}\;\mathrm{diameter}\right)\times 100\right)\;\;}{\mathrm{Basal}\;\mathrm{diameter}}$$

Carotid internal media thickness (CIMT) was evaluated using a 4.0 MHz probe (Koninklijke Philips N.V., Amsterdan, the Netherlands), according to the 2004 Mannheim Consensus. Briefly, with the patient in a supine position, primary transverse and longitudinal scans of the common carotid artery were performed, focusing on the posterior carotid wall at the carotid bifurcation and the common carotid artery. CIMT was measured approximately 1 cm from the bifurcation of the common carotid artery as the largest distance between the lumen–intima interface and the media–adventitia interface. The results are expressed as the mean of at least four measurements. The reproducibility of the measurements was validated by obtaining an acceptable correlation coefficient (>0.85) for interobserver reliability.

### 2.6. Balloon Angioplasty

The procedure of balloon angioplasty was performed as follows: An 18-gauge needle was placed into the blood vessel at the selected groin site, and an introducer was placed over the needle. Then, a flexible guide wire was advanced into the introducer and the guide was replaced with a 6-French introducer. Under fluoroscopic guidance, contrast medium was endovascularly injected in order to identify the artery trajectory and blocked vascular sites. Once the damaged vascular site was identified, two 0.014-French navigation guidewires and two 0.014-French support guidewires were introduced into the vessel; and they were advanced up to the blocked site. One 5-French catheter and one 3-French catheter were introduced, and blood from the site closest to the vascular obstruction was obtained. This was considered the preangioplasty sample (“baseline”). Then, a guidewire was advanced again, and when the guidewire was in place, an angioplasty balloon catheter was advanced and placed at to the site of lesion. Angioplasty was performed by balloon inflation against the blocking plaque located at the vascular wall; whole restoration of the blood flow was verified by injection of media contrast. The use of medicated stents was performed at the discretion of the attending physician. Thirty minutes after performing the balloon angioplasty, another catheter was advanced to the site closest to the angioplasty site and blood was collected. This was considered the postangioplasty sample (“reactive”). Finally, all of the guidewires were removed and the appropriate postoperative care was provided. 

### 2.7. Endothelial Precursor Cells Determination

Two 8 mL blood samples were collected from the femoral artery blood circulation, close to the atherosclerotic lesion site; one sample was obtained before angioplasty (“baseline”); and a second one after 30 min postangioplasty, in order to evaluate the immediate changes (“reactivity”) of the EPCs after angioplasty. For EPC isolation, Lymphoprep (Stem Cell Technologies, Vancouver, Canada) density gradient was used following standardized protocol. Blood samples were diluted in a 1:2 ratio with PBS and mixed. Then, the mixture was transferred to correspondent 15 mL tubes with Ficoll (density 1.077 g/mL), where two density gradients formed. Then, samples were centrifuged at 2000 rpm for 30 min. The mononuclear cell layer was collected with a Pasteur pipette and transferred to a new tube. After three washes with PBS, lymphocytes were counted and fixed in 4% paraformaldehyde and stored for a maximum of 12 h at 4 °C. Flow cytometry analysis (MACSQuant Analyzer 10—Miltenyi Biotec) was performed in a total of 1,000,000 events collected for each analysis, and corresponding isotype controls were used to set appropriate regions. EPC subpopulations were identified by their specific marker combination (Table 1), and they were reported as the percentage of the number of gated events. 

Subpopulations of EPCs of interest were the following immunophenotypes: CD45^+^CD34^+^CD133^+^; CD45^+^CD34^+^CD184^+^; CD45^+^CD34^+^CD133^+^CD184^+^; CD45^+^CD34^+^KDR^+^; CD45^+^CD34^+^KDR^+^CD133^+^; and CD45^+^CD34^+^KDR^+^CD184^+^.

### 2.8. Clinical Follow-Up

After the angioplasty procedure, all patients were followed up during the next 30 days; and the number of patients who required lower limb amputation because of unfavorable clinical evolution was registered.

### 2.9. Statistical Analysis

All continuous variables were expressed as the mean ± standard deviation (SD). Categorical measures were expressed as numbers (%). Data distributions for the variables were estimated using Kolmogorov–Smirnov test. Independent (1-tailed) T-test or Mann–Whitney test were applied for comparison between mean values, and the relation between variables was analyzed by the Spearman correlation test. Discrimination ability and association risk were evaluated through sensitivity, specificity, and relative risk (CI95%). All statistical analyses were performed using SPSS software, version 23.0 (SPSS Inc., Chicago, IL, USA) for Windows^®^, and a *p* value ≤0.05 was considered to be statistically significant.

## 3. Results

Twenty males, mean age 68 years old, with CLI and being candidates for balloon angioplasty, constituted the study population (Table 2). Half of them were smokers, while most prevalent comorbidities were diabetes mellitus and arterial hypertension. Vascular flow compromise was mostly expressed by the scores of Rutherford VI and WIFI I-III, as well as ABI 0.52 and FMD −2.27. Angioplasty was performed either at femoral + tibial and/or popliteal arteries (55%), or tibial alone and/or tibial + popliteal arteries (45%), with a technical success for all patients reported in this study. 

During a 30-day follow-up period after angioplasty, eight patients (40%) underwent major limb amputation, who were characterized by being a higher number of smokers and having a higher severity of vascular compromise, as depicted by higher WIFI and lower FMD. 

**Figure 1:**
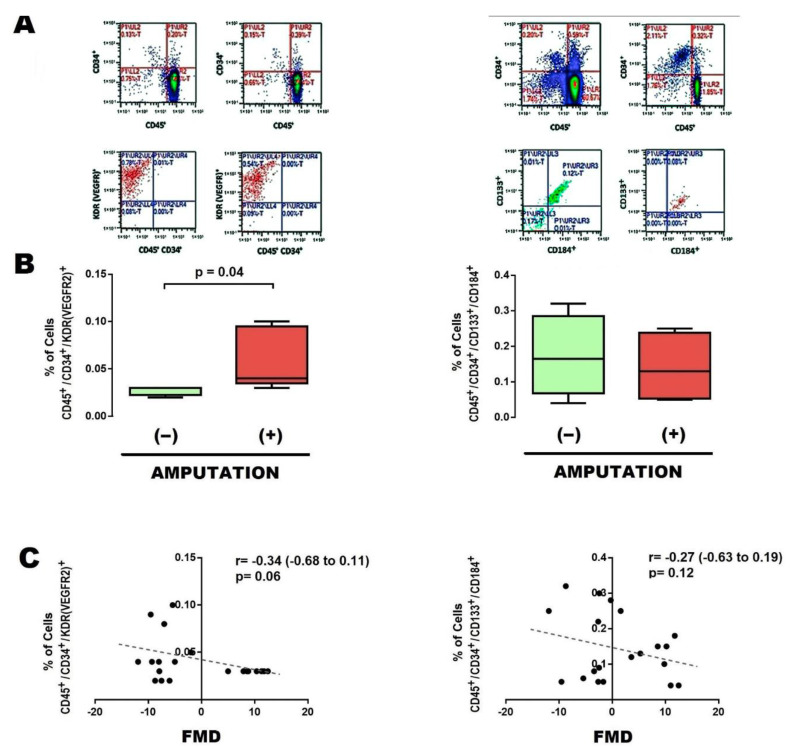
Baseline EPCs in patients with limb amputation. (**A**) Typical results of flow cytometry, with the markers used to identify EPCs in the preangioplasty phase, either in EPCs CD45^+^,CD34^+^,KDR [VEGFR2]^+^ (left panel) or CD45^+^,CD34^+^,CD133^+^,CD184^+^ (right panel). (**B**) Percentage of populations of EPCs CD45^+^,CD34^+^,KDR [VEGFR2]^+^ (left panel) and CD45^+^,CD34^+^,CD133^+^,CD184^+^ (right panel), present in blood circulation at a site near the vascular lesion, in cases that evolved without limb amputation (−) and with limb amputation (+). (**C**) Correlation between percentage of populations of EPCs vs. flow-mediated dilation (FMD). Comparisons were made with U-Mann–Whitney and Kruskall–Wallis, 1-way. Abbreviations: EPCs, endothelial progenitor cells; FMD, flow–mediated dilation.

**Figure 2:**
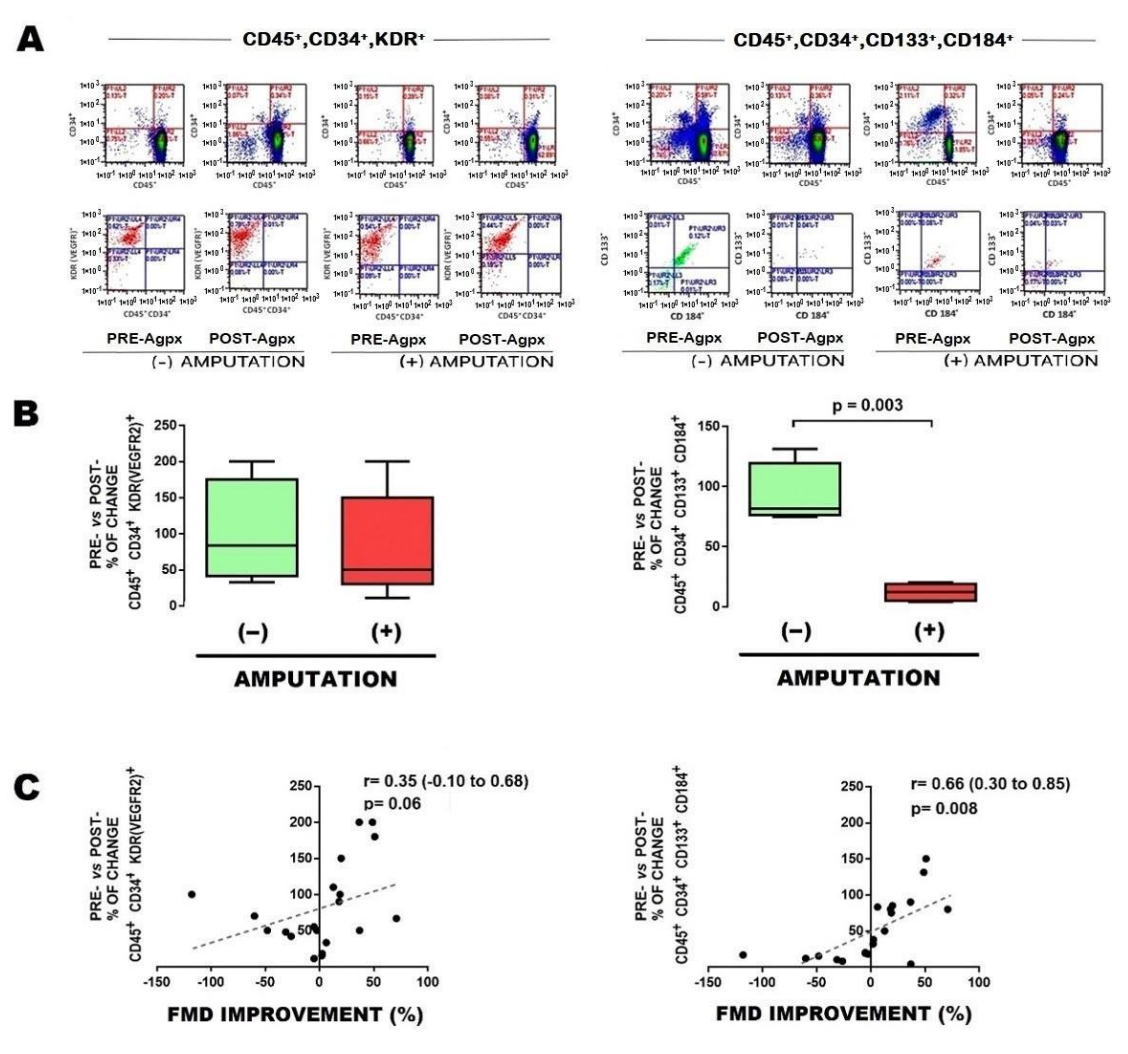
Postangioplasty changes in EPCs from patients with limb amputation. (**A**) Typical results of flow cytometry, with the markers used to identify EPCs, showing changes pre- vs. postangioplasty (PRE-Agpx vs. POST-Agpx) phases, either in EPCs CD45^+^, CD34^+^,KDR [VEGFR2]^+^ (left panel), or CD45^+^,CD34^+^,CD133^+^,CD184^+^ (right panel), in cases who evolved without limb amputation (−) and with limb amputation (+). (**B**) Graphics showing the percentage of change pre- vs. postangioplasty between cases who evolved without limb amputation (−) and with limb amputation (+). (**C**) Correlation between change in percentage of EPCs vs. change in flow-mediated dilation (FMD). Comparisons were made with U-Mann–Whitney and Kruskall–Wallis, 1-way. Abbreviations: EPCs, endothelial progenitor cells; PRE-Agpx, preangioplasty; POST-Agpx, postangioplasty; FMD, flow-mediated dilation.

In order to start characterizing the potential predictive role of EPCs, we determined the number of subpopulations of EPCs in blood sample obtained from the femoral arterial circulation, either before and after vascular angioplasty. Patients who further underwent major limb amputation showed an increased baseline (preangioplasty) number of EPCs CD45^+^CD34^+^KDR^+^ (Figure 1A [left] and Figure 1B [left]); as well as a postangioplasty reduction in EPCs CD45^+^CD34^+^CD133^+^CD184^+^ (Figure 2A [right], Figure 2B [right], and Table 2).

To further support the clinical implication of EPCs, correlation with FMD was determined. In general, the baseline subpopulation of EPCs CD45^+^CD34^+^KDR^+^ negatively correlated with FMD (Figure 1C [left]), whereas the change in EPCs CD45^+^CD34^+^CD133^+^CD184^+^ after angioplasty significantly correlated with FMD improvement (Figure 2C [right]). 

Finally, the role of EPCs as risk factors for major limb amputation was characterized, being specifically significant for EPCs CD45^+^CD34^+^KDR^+^ (Table 3: sensitivity 0.75, specificity 0.83, RR 4.5, *p* = 0.01). 

## 4. Discussion

Clinical demographic characteristics of the study population are comparable to other populations recruited in similar studies, such as Heinen Y et al. [28]; whereas a 30-day follow-up was considered because a significant number of limb amputations occur during this time period, and it might elicit appropriate comparisons with other studies similarly describing a 30-day follow-up [29,30].

We observed that factors such as smoker status, as well as vascular damage and endothelial dysfunction, as indicated by WIFI and FMD, were related to short-term major limb amputation after revascularization. These findings are consistent with other studies, highlighting their prognostic relevance [31,32,33]. 

Regarding characterization of the potential predictive role of EPCs for major limb amputation, some considerations should be stated: 1) Several immunophenotypes of EPCs were identified; however, we focused only in EPC subpopulations CD45^+^CD34^+^KDR^+^, which are considered a more mature and specialized immunophenotype of EPCs, and the immunophenotype CD45^+^CD34^+^CD133^+^CD184^+^ subpopulation of EPCs, considered less mature and less specialized. 2) We selected these immunophenotypes because of their particular relation with vascular damage and their reactivity to angioplasty, which was also observed during coronary angioplasty in a previous study from our group [28].

A main finding of the present study was the increased baseline (preangioplasty) number of circulating EPCs CD45^+^CD34^+^KDR^+^, obtained from blood flow at the proximity of vascular lesion, from patients who progressed to major limb amputation; suggesting that an increase in EPCs CD45^+^CD34^+^KDR^+^ indicates worse vascular prognosis, probably due to an impaired cellular ability prompting to repair advanced endothelial damage. This is consistent with previous studies showing increased circulating EPCs in acute myocardial infarction and stroke [29,30], and with the observation that the number of EPCs CD45^+^CD34^+^KDR^+^ was negatively related to FMD value, which confirms the potential interaction between EPCs CD45^+^CD34^+^KDR^+^ and endothelial damage and dysfunction. However, the role of EPCs CD45^+^CD34^+^KDR^+^ within endothelial dysfunction may be still under debate, since converse results have been obtained [34,35,36], probably due to differences in the design to explore this target. 

On the other hand, the lower ability to increase circulating EPCs CD45^+^CD34^+^CD133^+^CD184^+^ after angioplasty (“reactive”) was also related to major limb amputation, suggesting that an impaired reactive mobilization of EPCs CD45^+^CD34^+^CD133^+^CD184^+^ to the vascular injury is related to postangioplasty vascular recovery and may impact vascular prognosis. This last notion is supported by the observed changes in EPCs CD45^+^CD34^+^CD133^+^CD184^+^, which parallel FMD improvement [37,38]. Mobilization of EPCs has been reported to contribute to vascular repair of ischemic tissues. These cells are considered elements from vascular biology with potential clinical implications [27,39]. Consistently, a higher baseline number of EPCs CD45^+^CD34^+^KDR^+^ was found in coronary circulation from patients who would further develop cardiovascular adverse events after coronary angioplasty during one-year follow up; indicating that EPCs CD45^+^CD34^+^KDR^+^ may be related to a worse vascular prognosis at a coronary level [27]. 

Finally, further characterization of EPCs CD45^+^CD34^+^KDR^+^ was determined through their discrimination ability and association risk, which rendered a significant sensitivity of 0.75, specificity of 0.83, and RR of 4.5; suggesting that EPCs CD45^+^CD34^+^KDR^+^ may have predictive ability for major limb amputation, as well as their potential as a clinically useful cell biomarker of vascular damage and repair. Some limitations in the present study should be considered: (1) The low sample size; nevertheless, the aim of the present study was to explore the biological implication and clinical potential of EPCs as predictors of limb amputation. Then, considering EPCs as markers predicting limb amputation, our simple size renders a minimum beta-power of 0.8. (2) Time sequence for blood acquisition after angioplasty was performed, but determination of EPCs was shown only from 30 min postangioplasty. This time was selected from standardization experiments, however, a dynamic determination of EPCs would show a more reliable behavior of these biomarkers than a single determination time. (3) Due to the miscellaneous diseases from the study population, a multivariable test would have been desirable to analyze specific effects from EPCs; however, this would require a greater number of patients, and therefore, further studies in this translational field are warranted.

EPCs are a promising area of research for the treatment and prevention of vascular diseases, since these cells may play a role in maintaining the integrity of the vasculature by preventing the development of endothelial damage and atherosclerosis. EPCs are also being investigated as a potential tool for regenerative medicine, which has led to the development of EPC-based therapies for tissue regeneration and wound healing. In addition, EPCs are being investigated for use in cancer therapy. The field of EPC research is relatively new, and many questions remain to be answered. Nevertheless, the potential therapeutic benefits of EPCs in the treatment and prevention of vascular diseases are exciting and provide a promising area for future research. Further research is needed to fully understand the mechanisms underlying the effects of EPCs and to establish their safety and efficacy in clinical settings. The potential therapeutic benefits of EPCs in the treatment and prevention of vascular diseases, regenerative medicine, and cancer therapy provide a promising area for future research regarding these biomarkers.

In conclusion, EPCs are a population of stem cells that have the potential to repair and regenerate blood vessels. Their isolation, expansion, and therapeutic use have been studied in preclinical and clinical settings, and they are considered a promising strategy to improve the outcome of patients with vascular damage. However, more research is needed to fully understand the mechanisms of their effects and to establish their safety and efficacy in clinical settings.

## 5. Conclusions

In conclusion, EPCs were related with endothelial dysfunction, whereas a higher baseline number of EPCs CD45^+^CD34^+^KDR^+^, obtained from blood flow at the proximity of vascular lesion, as well as an impaired reactivity of EPCs CD45^+^CD34^+^CD133^+^CD184^+^ after angioplasty, showed a predictive ability for major limb amputation in patients with critical limb ischemia. 

## Figures and Tables

**Table 1:** Antibodies used for flow cytometry.

Antibody	Cell Marker	Fluorochrome/ Dilution	EPC Subpopulations Identified
CD45	Lymphocytes, endothelial, and/or vascular cells.	Vioblue 1:60	CD45^+^CD34^+^CD133^+^ CD45^+^CD34^+^CD184^+^ CD45^+^CD34^+^CD133^+^CD184^+^ CD45^+^CD34^+^KDR^+^ CD45^+^CD34^+^KDR^+^CD133^+^ CD45^+^CD34^+^KDR^+^CD184^+^
CD34	Endothelial and/or vascular cells	FITC 1:20	
KDR(VEGFR-2)	Membrane marker of endothelial cells	APC 1:60	
CD133	Endothelial progenitor cells	PE 1:50	
CD184	Hematopoietic stem cells and endothelial cells	PE-Vio770 1:20	

**Table 2:** Study population (*n* = 20).

	All (*n* = 20)	Non-Limb Amputation (*n* = 12)	Limb Amputation * (*n* = 8)	*p*-Value
Age (years old)	68.0 (64.7–76.5)	66.0 (55.0, 69.0)	68.0 (62.0, 70.5)	0.19
Smoking	10 (50.0)	2 (25.0)	8 (66.6)	**0.03**
Comorbidities				
*DM*	9 (45.0)	5 (41.6)	4 (50.0)	0.53
*DM + Hyp*	4 (20.0)	3 (25.0)	1 (12.5)	0.46
*DM + Hyp + CVD*	4 (20.0)	2 (16.7)	2 (25.0)	0.53
*DM + Hyp + CVD + Others*	3 (15.0)	2 (16.7)	1 (12.5)	0.66
Vascular profile				
*Rutherford*				
- *IV, V* - *VI*	3 (15.0) 17 (85.0)	1 (8.3) 11 (91.7)	2 (25.0) 6 (75)	0.34 0.34
*Wifi*				
- *I* - *II* - *III* - *IV*	6 (30.0) 2 (10.0) 8 (40.0) 4 (20.0)	6 (50.0) 2 (16.7) 3 (25.0) 1 (8.3)	0 (0.0) 0 (0.0) 5 (62.5) 3 (37.5)	**0.02** 0.34 0.11 0.15
*Ankle-to-Brachial Ratio*	0.52 ± 0.19	0.60 (0.50, 0.60)	0.53 (0.42, 0.67)	0.44
*Flow-Mediated Dilation*	−2.27 ± 8.98	7.66 (4.91, 11.3)	−9.2 (−10.6, −6.03)	**<0.001**
Angioplasty Site *Femoral + tibial and/or popliteal* 11 (55.0) 5 (40.0) 6 (75.0) 0.16 *Tibial alone and/or tibial + popliteal* 9 (45.0) 7 (60.0) 2 (25.0) 0.16 **Endothelial Progenitor Cells**				
CD45,CD34, KDR				
Pre-aptx	0.03 (0.03, 0.06)	0.03 (0.02, 0.03)	0.04 (0.03, 0.09)	**0.04**
Post-aptx	0.02 (0.15, 0.05)	0.02 (0.01, 0.03)	0.02 (0.01, 0.08)	0.21
% of change	66.7 (41.7, 150.0)	83.3 (41.7, 175.0)	50.0 (30.6, 150.0)	0.36
CD45,CD34,CD133,184				
Pre-aptx	0.16 (0.05, 0.23)	0.16 (0.06, 0.28)	0.13 (0.05, 0.23)	0.34
Post-aptx	0.02 (0.01, 0.14)	0.13 (0.05, 0.35)	0.01 (0.01, 0.01)	**0.04**
% of change	47.5 (7.9, 82.5)	81.7 (76.3, 119.3)	10.8 (4.3, 19.2)	**<0.01**

Results were expressed as median (p25-p75) or *n* (%). (*) All limb amputations were performed above the knee. CVD includes myocardial infarction and/or stroke. Others refers to CVD combined with cancer (breast or colon cancer). Abbreviations: DM, diabetes mellitus; Hyp, hypertension; CVD, cardiovascular disease; TcPO_2_, transcutaneous pressure of oxygen; Pre-aptx, preangioplasty; Post-aptx, postangioplasty.

**Table 3:** Associated risk.

EPC Subpopulation	Non-Limb Amputation (*n* = 12)	Limb Amputation (*n* = 8)	SE	SP	RR (CI95%) *p*-Value	
CD45,CD34, KDR (Pre-aptx)	2 (16.7)	6 (75)	**0.75**	**0.83**	**4.5 (1.2, 17.0)** ***0.01***	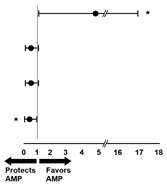
CD45,CD34,CD133,184 (Pre-aptx)	9 (75.0)	3 (37.5)	0.37	0.25	0.4 (0.1, 1.2) *0.11*	
CD45,CD34, KDR (% of change)	9 (75.0)	3 (37.5)	0.37	0.25	0.4 (0.1, 1.2) *0.11*	
CD45,CD34,CD133,184 (% of change)	9 (75.0)	2 (25.0)	0.25	0.25	**0.3 (0.1, 1.0)** ***0.04***	

Cutoffs: preaptx CD45, CD34, KDR: 0.03%; pre-aptx CD45, CD34, CD133, CD184: 0.15%; %change CD45, CD34, KDR: 66.6%; %change CD45, CD34, CD133, CD184: 75%. (*) = *p* < 0.05. Abbreviations: EPCs, endothelial progenitor cells; SE, sensitivity; SP, specificity; Pre-aptx, preangioplasty.

## Data Availability

Datasets analyzed or generated during this study can be requested from the authors.
